# Illumina sequencing of 15 deafness genes using fragmented amplicons

**DOI:** 10.1186/1756-0500-7-509

**Published:** 2014-08-09

**Authors:** Filip Van Nieuwerburgh, Sarah De Keulenaer, Joachim De Schrijver, Jo Vandesompele, Wim Van Criekinge, Paul J Coucke, Dieter Deforce

**Affiliations:** Laboratory for Pharmaceutical Biotechnology, Ghent University, Ottergemsesteenweg 460, Ghent, 9000 Belgium; NXTGNT, Ghent University, Ghent, Belgium; Center for Medical Genetics, Ghent University, Ghent, Belgium; Biobix, Laboratory for Bioinformatics and Computational Genomics, Ghent University, Ghent, Belgium

## Abstract

**Background:**

Resequencing of deafness related genes using GS FLX massive parallel sequencing of PCR amplicons spanning selected genes has previously been reported as a successful strategy to discover causal variants. The amplicon lengths were designed to be smaller than the sequencing read length of GS FLX technology, but are longer than Illumina sequencing technology read lengths. Fragmentation is thus required to sequence these amplicons using high throughput Illumina technology.

**Methods:**

We performed Illumina sequencing in 4 patients on 563 multiplexed amplicons covering the exons of 15 genes involved in the hearing process. After exploring several fragmentation strategies, the amplicons were fragmented using Covaris sonication prior to library preparation. CLC genomic workbench was used to analyze the data.

**Results:**

We achieve an excellent coverage with more than 99% of the amplicons bases covered. All variants that were previously validated using Sanger sequencing, were also called in this study. Variant calling revealed less false positive and false negative results compared to the previous study. For each patient, several variants were found that are reported by ClinVar as possible hearing loss variants.

**Conclusion:**

Migration from GS FLX amplicon sequencing to Illumina amplicon sequencing is straightforward and leads to more accurate results.

**Electronic supplementary material:**

The online version of this article (doi:10.1186/1756-0500-7-509) contains supplementary material, which is available to authorized users.

## Background

Hearing loss (HL) is quite common with one in 500 newborns having bilateral permanent sensorineural HL of more than 40 dB HL [1]. In more than 50% of the cases, hearing loss (HL) originates from mutations in one of the many genes related to the hearing process. In most cases, inherited HL is monogenic, but unfortunately it is an extremely heterogeneous trait, with over 60 implicated genes (Hereditary Hearing Loss Homepage; http://hereditaryhearingloss.org) making it a real challenge for molecular diagnostics.

Recently, we published a study using semi-automated PCR amplification and Roche GS FLX sequencing in order to screen 15 autosomal recessive deafness genes in 5 patients with congenital genetic deafness [2]. A total of 646 specific primer sets for all exons and most of the UTR’s of the 15 selected genes were designed. In this study, we analyzed the exact same PCR products using Illumina GAIIx sequencing. The main reason to investigate this, is the higher throughput of Illumina technology. This experiment compares Roche GS FLX and Illumina sequencing without changing any other variables. It also shows the possibility of performing Illumina sequencing on amplicons that were prepared for Roche GS FLX sequencing and shows the possibility of sequencing amplicons that are longer than the Illumina paired-end read length. Peer reviewed reports on sequencing of fragmented amplicons is scarce: A review by Mamanova *et al.* discusses target-enrichment strategies for next-generation sequencing. A method for generation sequencing of fragmented long range PCR amplicons is reported and the caveats are discussed [3]. Harismendy and Frazer report a method for improving sequence coverage uniformity of targeted genomic intervals amplified by long range PCR using Illumina sequencing-by-synthesis technology [4].

## Methods

### Patient material, primer design and amplicon PCR

For this study, no additional primer design or PCR was performed. PCR products were obtained from the previous study for 4 of the 5 HL patients. Patients 1–4 correspond to patients 1–3 and 5 in the previous study. Ethical approval was obtained for all patients during the previous study at the university of Antwerp ethical committee. The fifteen autosomal recessive deafness genes (TMPRSS13, TMI, TECTA, SLC26A4, PCDH15, OTOF, GJB2, ESRRB, ESPN, CDH23, MYO7A, PJVK (DFNB59), MYO15A, TMC1, TRIOBP) were selected, based on their reported high mutation frequency [5]. The 646 PCR products cover all the coding sequences (CDS) and most of the UTR’s of the 15 genes. All PCR reactions were performed as singleplex PCRs with the same PCR reaction conditions (See [2] for details). The used primer sequences are detailed in Additional file 1: Table S1. The average generated amplicon length over the 646 PCR’s is 319 bp, resulting in an aggregate target size of approximately 0.2 Mb. The used primers are modified at their 5′ end with a universal M13 linker sequence for GS FLX sequencing. For each patient, 563 PCR products were equimolarly pooled to obtain an equal representation of every amplicon in the pool. The rest of the 646 PCR products were left out of the pool because the PCR product was exhausted in the previous study (detailed in Additional file 1: Table S1).

### Illumina sequencing

For Illumina GAIIx paired-end sequencing (2x100 bp) of amplicons bigger than 200 bp, these amplicons need to be fragmented. It is crucial that this fragmentation occurs as random as possible, to obtain an even read depth across the amplicons. Several fragmentation strategies were explored. One strategy was to concatenate the PCR products before fragmentation with Fast-Link DNA Ligation Kit (Epicentre Biotechnologies). The idea was to create longer DNA strands which might be easier to fragment randomly. This procedure requires substantially more DNA, takes longer, requires more hands on work, is more expensive and in the end provided no advantage over direct fragmentation of the amplicons in terms of coverage. For this reason, the data of these analyses are not shown in this report. For the fragmentation itself, we compared fragmentation with NEBNextTM dsDNA Fragmentase (NEB) with fragmentation using the Covaris S2. The distribution of the fragment lengths was much smaller with the Covaris protocol. Furthermore, the enzymatic fragmentation is not robust and DNA-concentration, enzyme concentration, incubation time and temperature should be tightly controlled. Even then, the yield of the desired fragments is low. Finally, the samples were fragmented using the Covaris S2 with following settings: Duty Cycle: 5% - Intensity: 5 - Cycles per burst: 200 - Time: 12 minutes. The fragments were used in a standard Illumina genomic sample preparation, cluster generation and 2x100 bp paired-end sequencing on an Illumina GAIIx sequencer.

### Data analysis

Using Cutadapt ( http://code.google.com/p/cutadapt/), the sequences were trimmed for the M13 linker sequences. Using an in house developed perl script, the reads were trimmed when the average quality in a sliding window of 10 bp falls below 25 and filtered for sequences shorter than 30 bp after trimming. The remaining sequences were analyzed using CLC genomics workbench version 6.5. The reads were paired end mapped to the haploid human reference sequence hg 19 (GRCh37) using a mask for the target regions of the used amplicons. Single nucleotide variants and short insertions and deletion were called using the CLC quality-based variant detection tool. Reads that did not map uniquely and broken pairs were ignored. Only variants with a coverage of at least 10 and a minimum variant frequency of 35% were called. The requirement that variants should be present more or less equally in forward and reverse reads was not used because this lead to too many false negatives. We explored the advantage of paired end (PE) mapping *versus* single end (SE) mapping: On average SE mapping results in 25% more called variants. Manual evaluation by comparing the PE and SE mappings on a subset of these extra variants revealed that most of these variants are likely to be false positives due to incorrect mapping of e.g. short SE reads. SE mapping is less stringent compared to PE mapping. In an amplicon sequencing setting, incorrect mappings can be propagated due to the many duplicate reads originating from identical amplicons, leading to a substantial increase of the number of incorrectly called variants. The paired-end variants were annotated using CLC and the ClinVar [6] database (ftp.ncbi.nih.gov/snp/organisms/human_9606/VCF/clinvar_00-latest.vcf.gz 05 Nov 2013 10:27:36) to find variants that might be involved in hearing loss. The amino acid changes caused by the variants were also calculated using CLC.

## Results

### Coverage

The number of generated raw paired-end 2x100bp reads for patient 1 to 4 was 12.8 × 10^6^, 8.6 × 10^6^, 3.2 ×10^6^ and 3.0 × 10^6^ respectively. Trimming for the M13 sequence, trims approximately 5% of the bases. After the subsequent quality trimming and filtering, 11.9 × 10^6^, 7.9 × 10^6^, 2.9 × 10^6^ and 2.8 x10^6^ reads remained with an average length of 85 bp. These reads have been deposited in the EMBL-EBI European Nucleotide Archive (ENA) with study accession PRJEB6747 and is available at http://www.ebi.ac.uk/ena/data/view/PRJEB6747. The percentage of mapped reads and the resulting coverage and depth is shown in Table 1.

**Table 1:** **Coverage results are shown for paired end mapping**

	Patient 1	Patient 2	Patient 3	Patient 4
**Number of raw Illumina GAIIx reads**	12.81E + 06	8.56E + 06	3.15E + 06	3.03E + 06
**Number of trimmed, quality filtered reads**	11.92E + 06	7.88E + 06	2.88E + 06	2.79E + 06
**Mapped reads - Single end**	98.77%	98.05%	98.82%	99.29%
**Mapped reads - Paired end**	99.02%	98.35%	99.14%	99.57%
**(properly paired)**	(97.71%)	(95.97%)	(96.82%)	(98.44%)
**Completely covered amplicons**	557/563	554/563	560/563	560/563
**Average depth**	13064	8661	3329	3270
**% of amplicon bases with depth > 0**	99.42%	99.30%	99.36%	99.55%
**% of amplicons bases with depth > 5**	98.68%	97.94%	99.17%	99.35%

Results for single end mapping are practically equal.

### Called variants

On average 172 variants were called in the 4 patients. The called variants can be found in Additional file 2: Table S2. The number of snps and indels called by CLC on the Illumina data and the numbers of snps and indels previously called by the VIP pipeline on the GS FLX data can be compared in Additional file 3: Table S3. All 5 variants that were previously found by the VIP pipeline and validated using Sanger sequencing, were also called by CLC in the Illumina data. The variants that were annotated by ClinVar as deafness related or as highly penetrant, can be found in Additional file 4: Table S4.

## Discussion

We performed Illumina sequencing on multiplexed amplicons that were designed for GS FLX sequencing. Using the exact same PCR products, we achieve an excellent coverage with more than 99% of the amplicons bases covered and around 98% of the bases covered with a depth greater than 5. This setup is not ideal to evaluate the performance of Illumina sequencing because the amplicons were generated using Roche specific fusion primers and the amplicons therefore contain uninformative sequences that will be sequenced by the Illumina technology. Furthermore, the amplicons are larger than the 200 bp, making it necessary to fragment the amplicons before they are sequenced. It is expected that the performance of Illumina sequencing should only increase when the amplicons would be generated with analogous primers that do not contain the Roche GS FLX adapter sequences. Nevertheless, we demonstrate that excellent results can be obtained in this setting with a simple Covaris sonication and a standard Illumina sample prep. A higher sequencing depth can be obtained at a lower cost compared to GS FLX sequencing. Furthermore, Sanger sequencing of homopolymer containing regions is not necessary because Illumina sequencing can correctly sequence these regions. Only about half the number of variants is called by CLC on the Illumina dataset compared to the VIP pipeline on the 454 dataset. All 5 deafness associated variants that were previously confirmed by Sanger sequencing were present in the CLC variant calls, while the unconfirmed variant was not present. This hints at the fact that the CLC variants contain less false positives and false negatives compared to the VIP variants. Several of the variants called by CLC are annotated as hearing loss related or as being highly penetrant and deleterious. The hearing loss related variants from all patients were checked using dbSNP [7] and a literature study. For patient 3, we also checked all variants that were marked as highly penetrant, because no definitive causal variants were found for this patient in the previous study. All variants had a minor allele frequency that is too high to be a causal variant or were reported in deafness related publications as neutral changes [8–11].

## Conclusion

Sequencing with Illumina technology of amplicons that were designed for Roche GS FLX sequencing results in excellent coverage after simple fragmentation of the amplicons using a Covaris sonication. All exons of 15 deafness related genes were more than 99% covered, leading to less false positive and false negative called variants. For each patient, several variants were found that are reported by ClinVar as possible hearing loss variants. Migration from GS FLX amplicon sequencing to Illumina amplicon sequencing is straightforward and leads to more accurate results.

## Consent

Written informed consent was obtained from the patients for publication of these findings in scientific journals. A copy of the written consent is available for review by the Editor of this journal.

## Electronic supplementary material

Additional file 1: Table S1: Primer sequences covering 15 deafness genes. (XLS 470 KB)

Additional file 2: Table S2: Called variants on 15 deafness genes. (XLSX 180 KB)

Additional file 3: Table S3: Comparison number of variants between GS FLX and Illumina data. (DOCX 16 KB)

Additional file 4: Table S4: "Variants annotated by ClinVar as deafness related". (XLSX 70 KB)
