# Supplementary material for: Illumina sequencing of 15 deafness genes using fragmented amplicons
Source: BMC Res Notes. 2014 Aug 9;7:509. doi: 10.1186/1756-0500-7-509 (PMC4266979; doi:10.1186/1756-0500-7-509)
Supplement: Supplementary file 3 — Additional file 3: Table S3: Comparison number of variants between GS FLX and Illumina data. (DOCX 16 KB) [file 13104_2014_3070_MOESM3_ESM.docx]

***Supplementary Table 3***

| **Number of variants** | **Patient 1** | **Patient 2** | **Patient 3** | **Patient 4** |
| --- | --- | --- | --- | --- |
| **CLC** | 169 | 146 | 184 | 190 |
| **VIP** | 361 | 339 | 404 | 330 |
| **Common CLC/VIP** | 144 | 126 | 171 | 154 |
| **Unique CLC** | 25 | 20 | 13 | 36 |
| **Unique VIP** | 217 | 213 | 233 | 176 |

Number of variants called by CLC software on Illumina data and VIP pipeline on GS FLX data.
